# Espresso Coffee Consumption and Risk of Coronary Heart Disease in a Large Italian Cohort

**DOI:** 10.1371/journal.pone.0126550

**Published:** 2015-05-06

**Authors:** Sara Grioni, Claudia Agnoli, Sabina Sieri, Valeria Pala, Fulvio Ricceri, Giovanna Masala, Calogero Saieva, Salvatore Panico, Amalia Mattiello, Paolo Chiodini, Rosario Tumino, Graziella Frasca, Licia Iacoviello, Amalia de Curtis, Paolo Vineis, Vittorio Krogh

**Affiliations:** 1 Epidemiology and Prevention Unit, Fondazione IRCCS Istituto Nazionale dei Tumori, Milan, Italy; 2 Human Genetics Foundation, Turin, Italy; 3 Unit of Cancer Epidemiology, University of Turin and Center for Cancer Prevention, Turin, Italy; 4 Molecular and Nutritional Epidemiology Unit, Cancer Research and Prevention Institute (ISPO), Florence, Italy; 5 Department of Clinical and Experimental Medicine, University of Naples Federico II, Naples, Italy; 6 Department of Medicine and Public Health, Second University of Naples, Naples, Italy; 7 Cancer Registry, Department of Prevention, Provincial Health Centre 7, Ragusa, Italy; 8 Laboratory of Molecular and Nutritional Epidemiology. Department of Epidemiology and Prevention, IRCCS Istituto Neurologico Mediterraneo, Pozzilli, Italy; 9 School of Public Health, Imperial College, London, United Kingdom; University College London, UNITED KINGDOM

## Abstract

**Background:**

The relationship between coffee consumption and coronary heart disease (CHD) has been investigated in several studies with discrepant results. We examined the association between Italian-style (espresso and mocha) coffee consumption and CHD risk.

**Methods:**

We investigated 12,800 men and 30,449 women without history of cardiovascular disease recruited to the EPICOR prospective cohort study. Coffee consumption was assessed at baseline. In a random sub-cohort of 1472 subjects, plasma triglycerides, and total, LDL and HDL cholesterol were determined to investigate the effect of coffee consumption on plasma lipids.

**Results:**

After a mean follow up of 10.9 years, 804 cases of CHD (500 acute events, 56 fatal events and 248 revascularizations, all first events) were identified. Multivariable adjusted hazard ratios for CHD were: 1.18 (95% CI 0.87–1.60) for drinking 1–2 cups/day, 1.37 (95% CI 1.03–1.82) for >2–4 cups/day and 1.52 (95% CI 1.11–2.07) for over 4 cups/day (*P* trend <0.001) compared to reference (<1 cup/day). Plasma triglycerides, and total, LDL and HDL cholesterol did not vary significantly (ANOVA) with coffee consumption.

**Conclusion:**

Consumption of over 2 cups/day of Italian-style coffee is associated with increased CHD risk, but coffee consumption was not associated with plasma lipid changes, so the adverse effect of consumption appears unrelated to lipid profile.

## Introduction

Coffee, one of the most widely-consumed beverages in the world, is extracted from the roasted seeds (beans) of *Coffea sp*., an evergreen plant native to Africa. The composition of a coffee beverage varies in relation to the species, the variety, the roast, and the preparation method. Most coffees purchased in Europe and North America are blends of beans from the species *robusta* and *arabica*, which have different chemical profiles. *Robusta* beans contain more caffeine and chlorogenic acids, and less lipids than *arabica* [1–3]. Methods of preparing coffee vary widely with geography and culture and can also result in variation in the chemical profile of the beverage, particularly as regards the content of lipids and other phytochemicals [4]. In Italy, coffee in bars is usually made by the espresso method in which high pressure hot (about 90°C) water is passed through 5–7 g of finely-ground powder to produce a 30 ml serving. The other traditional way of making coffee in Italy, particularly in the home, is to use a mocha: a three-part aluminum or stainless steel coffee-maker in which close-to-boiling water is forced up through the coffee into the top of the pot [5].

The relation of coffee consumption to coronary heart disease (CHD) has been extensively studied. Some studies suggest that coffee consumption is not a major risk factor for CHD [6–8]. Other studies have found that heavy/moderate coffee consumption is associated with increased risk of CHD in men [9,10], while another study reported that male coffee consumers without a family history of CHD were at reduced risk [11]. The most recent meta-analysis of cohort and case-control studies found discordant results on coffee consumption and CHD [12–14]. Most studies in this area investigated the consumption of either boiled unfiltered coffee or filtered coffee. Passing coffee through paper filters removes the lipids, notably the diterpene-fatty acid esters, which have been implicated in the increased total and low-density lipoprotein (LDL) cholesterol reported associated with coffee consumption [15].

To our knowledge few case-control studies have investigated the effects of Italian-style coffee consumption on CHD risk. One study found increased risk of acute myocardial infarction for heavy consumers [16]; while another study found that, in women, consumption of Italian-style coffee was associated with a non-significant increase in CHD risk [17]. A prospective study on Italian post myocardial infarction patients found no association between moderate coffee intake and subsequent cardiovascular events [18]. Another Italian study found that espresso and mocha coffee consumption had no effect on blood levels of cholesterol (total, LDL and HLD) or triglycerides in healthy young men over a six-week period [5]. By contrast, a meta-analysis found that coffee was directly related to increased total cholesterol, LDL-cholesterol and triglycerides when consumed boiled or non-filtered [19].

The aim of the present study was to prospectively investigate the relationship between the consumption of Italian-style coffee and the incidence of CHD in a large cohort of Italian men and women recruited in the EPICOR study [20]. The effect of coffee consumption on plasma triglycerides and cholesterol was also investigated in a random subsample of the cohort with plasma determinations available.

## Methods

### Study population and design

EPICOR is a prospective cohort study to investigate the risk of cardiovascular disease in the five centers (Varese, Turin, Florence, Naples, Ragusa) of EPIC Italy [21,22]. From 1993 to 1998 47,021 volunteers (14,863 men; 32,158 women) were recruited to EPIC Italy. The present EPICOR cohort consists of these volunteers, excluding 776 with a prior history of stroke or myocardial infarction.

Volunteers who did not complete dietary or lifestyle questionnaires (*n* = 907) were also excluded. To reduce the impact of implausible extremes we also excluded those in whom the ratio of total energy intake to basal metabolic rate was at either end of the distribution (cutoffs first and last half-percentiles; *n* = 802). Further persons were excluded for missing values of covariates (n = 1261) and implausible coffee consumption (over 15 cups/day; n = 16).

A total of 43,249 volunteers age 35–74 years, mean follow-up 10.9 years (471,139 person years) was included in the analyses. In a subset of 1472 randomly selected for a case-cohort study nested in the EPIC population, Florence excluded, and for whom plasma lipids were available, we assessed the relation of plasma lipids to coffee consumption. The study complies with the declaration of Helsinki and the study protocol was approved by the ethics committee of the Human Genetics Foundation, Turin. Participants gave informed consent to use clinical data for research.

### Dietary assessment

Participants completed a validated [23] semi-quantitative food frequency questionnaire (FFQ) which investigated food intake over the preceding year. The FFQ asked the number of times for each food item was consumed (per day, week, month or year) from which the frequency of consumption was calculated. The quantity of food consumed was assessed by asking the participant to select an image of a food portion or a predefined standard portion when no image was available. The estimated frequencies and quantities of each food were linked, using specifically designed software [21], to Italian Food Composition Tables [24] to obtain estimates of daily intakes of macronutrients, micronutrients and energy. The FFQ enquired about coffee consumption, with quantities estimated in terms of standard espresso cups (30 ml). Cappuccino and café latte were assumed to contain 20% coffee and 80% milk.

### Assessment of lifestyle and anthropometric factors

Information about medical history, physical activity, smoking, education, and other socioeconomic variables was collected using a standardized questionnaire. Height, weight and blood pressure were measured at enrollment using standardized procedures [25]. Hypertension was ascertained from self-reported treatment or measured pressure (systolic ≥140 mmHg, diastolic ≥90 mmHg). Body mass index (BMI) was calculated as weight (kg) divided by height (m) squared. Hyperlipidemia and diabetes were ascertained from self-reported diagnosis and treatment. Total physical activity was estimated by questions about average times spent in specific activities. Volunteers were classified in inactive, moderately inactive, moderately active and active [26].

### Biological samples

A 30ml fasting blood sample was collected using a standardized protocol, and stored in liquid nitrogen at 196°C [27]. Total cholesterol, HDL cholesterol and triglycerides were measured in plasma samples using commercial enzyme colorimetric kits (Instrumentation Laboratory, Milan, Italy) and an automatic analyzer (IL 350). Coefficients of variation (CV) for high level external standards were 5.5% for cholesterol, 5.0% for triglycerides, and 6.1% for HDL cholesterol. Corresponding CVs for low level external standards were 5.8%, 7.9% and 7.0%; corresponding CVs for an in-house plasma pool were 2.6%, 3.5% and 5.3%. LDL cholesterol was calculated using the Friedewald formula [28].

### Morbidity and mortality follow up

During follow-up, electronic hospital discharge records and mortality files were linked to the study database to identify incident and fatal cases of CHD. Case definition was based on International Classification of Diseases (ICD) 9th edition codes (410–414) and procedure codes for coronary revascularization; each case was verified by checking that the diagnosis was consistent with examinations and procedures performed (particularly percutaneous transluminal coronary angioplasty and coronary artery bypass surgery). If records were not exhaustive or information was discordant, clinical records were accessed and checked; CHD was considered verified when acute myocardial infarction, acute coronary syndrome, or coronary revascularization was present. All cases were censored at the date of the first event and were cross-checked with mortality files to identify fatal and nonfatal cases (the later defined as alive 28 days after diagnosis).

CHD deaths were identified in the mortality database by ICD 10th edition codes: I20- I25, R96, and R99, and when codes E10-E14, I10-I13, I30, I31, I33-I38, I40, I42, I44-I51, I70-I74, and I77 were reported with I20-I25 as associated conditions. Fatal cases were confirmed by consulting death certificates.

### Statistical Analysis

Coffee consumption was categorized as <1 cup/day (non consumers and occasional drinkers), 1–2 cups/day (light drinkers), > 2–4 cups/day (moderate drinkers) and >4 cups/day (heavy drinkers). Baseline characteristics of subjects, according to categories of coffee intake, were summarized as means and standard deviations (continuous variables) or frequencies (categorical variables). Differences across categories were examined by ANOVA for continuous variables and χ^2^-test for categorical variables. Follow-up was time from enrolment to CHD diagnosis, death, loss to follow-up, or end of follow-up, whichever came first. End of follow-up was December 31 of the following years: 2006 for Varese and Naples, 2003 for Florence, 2008 for Turin, and 2007 for Ragusa. Multivariate Cox proportional hazard models were used to investigate the association between coffee consumption and CHD events, with age as primary time-dependent variable. Model 1 was the crude model adjusted for sex, age, and stratified by center. Model 2 was additionally adjusted for hypertension (yes/no), diabetes (yes/no), hyperlipidemia (yes/no), smoking status (current/former/never) and intensity (pack-years), education (≤8 years, >8 years), alcohol consumption (0, up to 12, >12 g/day women; 0, up to 24, >24 g/day men), tea consumption (0, up to 150 ml/day; ≥150 ml/day), BMI (<25, 25–30, >30 kg/m^2^), and physical activity (ordinal) as well as waist circumference, nonalcoholic energy intake, fruit and vegetables and saturated fatty acids intake (all continuous). HRs of CHD events, with 95% confidence intervals (CI), were estimated for categories of coffee consumption, with <1 cup/day as reference. Linear trends across categories were tested treating each category as a continuous variable in the Cox model. We also ran continuous models to investigate a dose-dependent effect for one cup increments. Interactions of coffee consumption with alcohol intake, smoking status, sex and hypertension were tested. Models with and without multiplicative interaction terms were compared using the likelihood ratio test. P values <0.05, 2-sided tests, were considered significant.

We also examined the association of coffee intake with plasma levels of total, LDL and HDL cholesterol and triglycerides in 1472 volunteers with data available. Differences in mean levels of plasma lipids between categories of coffee consumption were tested by analysis of variance after adjusting for the covariates included in model 2 (above). All analyses were performed using STATA version 11.2 (College Station, TX).

## Results

During a mean follow-up of 10.9 years, 804 cases of CHD (500 acute events, 56 fatal events and 248 revascularizations) were identified. Baseline characteristics of the cohort are presented by levels of daily coffee consumption in Table 1. Those who drank more than 4 cups of coffee per day were younger, more likely to smoke, have high energy intake, have high fruit and vegetables intake and have low tea intake; they were also less likely to have normal weight (BMI ≤25 k/m^2^) and hypertension.

**Table 1:** Baseline characteristics of 43,249 EPICOR participants by coffee consumption.

	<1 cup/day	1–2 cups/day	>2–4 cups/day	>4 cups/day
Participants (N)	4073	10179	19612	9385
BMI <25 (%)	52.1	46.1	44.6	41.1
Education >8 years (%)	44.8	50.4	49.1	49.0
Alcohol (g/day)	10.8 (16.9)	12.9 (16.9)	12.9 (16.3)	11.4 (15.9)
Hypertension, yes (%)	38.1	42.7	39.0	33.9
Systolic blood pressure (mmHg)	129.5 (18.4)	130.9 (18.5)	129.5 (18.0)	127.4 (17.6)
Diastolic blood pressure (mmHg)	81.6 (9.9)	82.4 (10.2)	81.8 (10.1)	81.0 (10.0)
Smoking, yes (%)	15.6	19.2	27.5	41.9
Diabetes, treated* (%)	1.2	0.9	0.9	0.9
Hyperlipidemia, treated * (%)	3.1	3.7	2.7	2.3
Non-alcohol energy (kcal/day)	2111.6 (656.7)	2121.5 (633.0)	2234.6 (643.9)	2419.0 (699.7)
Age (years)	50.3 (8.1)	51.0 (7.9)	50.3 (7.7)	48.8 (7.4)
Waist (cm)	82.9 (12.0)	84.3 (11.9)	84.2 (11.7)	84.5 (12.0)
BMI (kg/m^2^)	25.3 (4.1)	25.9 (4.2)	26.0 (4.0)	26.3 (4.1)
Inactive (%)	28.3	33.1	28.4	27.3
Saturated fat (g/day)	28.3 (11.2)	28.6 (10.8)	30.9 (11.1)	34.5 (12.4)
Tea (ml/day)	86.0 (137.2)	46.7 (88.5)	34.2 (69.1)	30.3 (70.1)
Fruit and vegetables (g/day)	547.0 (278.2)	536.2 (255.9)	540.4 (247.8)	559.7 (264.3)

Values are means with standard deviations in parentheses, unless otherwise indicated. All variables differ significantly (P <0.001) between categories of coffee consumption except diabetes.

* Self-reported on lifestyle questionnaire.

In the multivariable models (Table 2) we found that risk of CHD was significantly higher than reference (<1 cup/day) for coffee consumption above 2 cups/day, with HRs of 1.37 (95% CI 1.03–1.82) for >2–4 cups/day and 1.52 (95% CI 1.11–2.07) for >4 cups/day (P trend <0.001).

**Table 2:** Hazard ratios (HRs) for developing CHD in relation to daily coffee intake.

	Coffee intake				One cup/day increment	P trend
Cups/day	<1	1–2	>2–4	>4		
Cases/person-years	55/44900	169/111648	374/212221	206/102370	804/471139	
HRs (95%CI) *	1.00	1.21 (0.89, 1.64)	1.51 (1.14, 2.01)	1.99 (1.48, 2.68)	1.13 (1.08, 1.17)	<0.001
HRs (95%CI) ^†^	1.00	1.18 (0.87, 1.60)	1.37 (1.03, 1.82)	1.52 (1.11, 2.07)	1.06 (1.01, 1.10)	0.002

* Model 1: adjusted for sex and age at recruitment, stratified by center.

^†^ Model 2: model 1 with additional adjustments for non-alcohol energy intake, hypertension (yes/no), diabetes (yes/no), hyperlipidemia (yes/no), alcohol intake (0, up to 12, >12 for women; 0, up to 24, >24 for men), fruit and vegetables intake, tea consumption (0, up to 150 ml/day, >150 ml/day), saturated fatty acid intake, smoking status, smoking pack-years, education (≤8 years, >8 years), BMI (≤25, 25–30, >30), waist circumference (cm), and physical activity. One cup = 30 ml.

The association between coffee and CHD was independent of sex (P interaction 0.873), age (P interaction 0.058), smoking status (P interaction 0.596), hypertension (P interaction 0.897), BMI (P interaction 0.412) and alcohol consumption (P interaction 0.544). For the 1472 volunteers with laboratory data, triglycerides, and total, LDL and HDL cholesterol were unrelated to coffee consumption (Table 3).

**Table 3:** Multivariable-adjusted means* (standard error in parentheses) of triglycerides and total, LDL and HDL cholesterol, by categories of coffee consumption, in 1472 randomly selected EPICOR volunteers.

	Total cholesterol, mg/dL	LDL mg/dL	HDL mg/dL	Triglycerides mg/dL
<1 cup/day	232.9 (4.3)	145.5 (3.7)	60.2 (1.2)	135.9 (7.0)
1–2 cups/day	233.7 (2.6)	145.3 (2.2)	59.1 (0.7)	147.0 (4.3)
>2–4 cups/day	234.9 (1.7)	147.8 (1.5)	58.2 (0.5)	144.9 (2.8)
>4 cups/day	233.9 (2.5)	148.1 (2.1)	55.8 (0.7)	149.7 (4.1)
P value^†^	0.780	0.583	0.963	0.760

* Adjusted for sex, age at recruitment, non-alcohol energy intake, hypertension (yes/no), diabetes (yes/no), hyperlipidemia (yes/no), alcohol intake (0, up to 12, >12 for women; 0, up to 24, >24 for men), saturated fatty acid intake, smoking status, education (≤8 years, >8 years), BMI (≤25, 25–30, >30), waist circumference (cm), and physical activity.

^†^ ANOVA.

## Discussion

In this study the risk of CHD was significantly greater than reference (<1 cup/day) for those whose intake of Italian-style coffee was greater than 2 cups per day. We also found, in a randomly selected sub-cohort, that coffee consumption was not significantly associated with plasma levels of total, LDL or HDL cholesterol, or triglycerides. These results are consistent with those of an Italian case-control study published in 2001 [16,17] which found that CHD risk was increased in heavy consumers of Italian-style coffee. Similarly an Italian cohort study of 11,231 patients with recent myocardial infarction found no association between moderate coffee intake and subsequent cardiovascular events over 3.5 years of follow-up [18]. Other studies have found discordant relations between filtered or unfiltered coffee consumption and CHD risk. A case-control study reported no association between either caffeinated and decaffeinated coffee and risk of myocardial infarction [8]; and in a Swedish case-control study coffee consumption was inversely related (not significant) to risk of myocardial infarction in women [7]. Another case-control study on espresso coffee and myocardial infarction in Portuguese men found a significant inverse association for men without a family history of acute myocardial infarction (AMI), but a direct non-significant association for men with a first degree relative who had suffered AMI [11]. No evidence of an adverse association between coffee and CHD for men or women was found in a large cohort study [6], while a cohort study on middle-aged Finnish men suggested increased CHD risk related to consumption of over 800 ml/day of boiled or filtered coffee [9] and a large prospective cohort study found a lower incidence of CHD morbidity, with U shaped relation [29].

A meta-analysis of 13 case-control studies and 10 cohort studies found a significant association between high coffee consumption and CHD in the case-control studies, but no significant association between daily coffee consumption and CHD on long-term follow-up of cohort studies [13]. A recent meta-analysis of 5 cohort studies indicated a J-shaped relationship between coffee consumption and heart failure incidence, with a modest inverse association for moderate consumption [12]. However a meta-analysis of 21 prospective cohort studies found no increase in risk of CHD in any coffee consumption category, and a lower risk for moderate coffee consumption in women [14].

To our knowledge the present study is the first cohort study to specifically investigate the effects of Italian-style coffee on risk of CHD in a healthy population. Most previous studies have been concerned with filtered or boiled coffee, which are the most popular preparation methods in the USA and Scandinavian countries, respectively [30]. The preparation method can affect the concentrations of diterpenes and caffeine. The content of cafestol and kahweol is around 7.2 mg/cup of each substance in boiled coffee, 2.3 mg/cup of each substance in mocha coffee, and around 1.0 mg/cup of each substance in espresso coffee, but is only 0.02 mg/cup in filtered coffee [31]. Boiling results in high lipid concentrations because of the higher water temperature and longer contact time. Filtration through paper removes most lipids, while for Italian-style coffee the water has a short contact time with the solid coffee explaining the lower lipid concentrations than boiled coffee, but considerably higher concentrations than paper-filtered coffee [31]. In fact, although the diterpene concentration of Italian-style coffee is quite high [31], mean cup size in much lower than in many European countries so the dose would not be high enough to affect plasma cholesterol levels, consistent with the findings of our study.

A randomized trial on the effect of Italian-style coffee on serum cholesterol in young men did not show any alteration of the cholesterol or lipoprotein profile [5]. Nevertheless a meta-analysis of 12 randomized controlled trials found that coffee consumption was associated with significant increases in total and LDL cholesterol and triglycerides, but that the effect was greater for unfiltered coffee [19].

A standard cup (30 ml) of espresso contains about 100 mg of caffeine [32], while a 225 ml cup of boiled or filtered coffee contains about 135 mg of caffeine [30]. The effect of caffeine on the cardiovascular system depends on various factors including metabolic status and presence of illness [33]. The caffeine in coffee has variable effects on blood pressure: sporadic consumption by normotensive and hypertensive individuals has a pressor effect, but long-term consumption does not further increase blood pressure in hypertensive individuals [1,2]. In the EPIC-Florence cohort study coffee was significantly and inversely related to both systolic and diastolic blood pressure among women in the highest quintile of consumption (>3 cups/day) [34]. Caffeine is absorbed rapidly after ingestion: plasma levels peak at 30 min, with 75% of the maximum level reached after 15 min, and total absorption from the gut complete in about 45 minutes [35].

Caffeine is metabolized by cytochrome P4501A2 (CYP1A2) in the liver. Individuals who are homozygous for the CYP1A2*1A allele are rapid caffeine metabolizers, while carriers of CYP1A2*1F are slow metabolizers. In a case-control study, high coffee intake was associated with increased risk of non-fatal myocardial infarction only in individuals with the CYP1A2*1F allele [36], suggesting that caffeine is involved in the coffee-CHD association. The Harvest study [37] found that increased risk of hypertension was associated with coffee intake only among individuals with the slow allele (59% of the population); whereas for rapid metabolizers, coffee consumption was inversely related to hypertension.

Other studies have found a direct relation between homocysteine levels and filtered and unfiltered coffee intake [38,39]. A double-blind crossover study on healthy subjects found that caffeinated coffee consumption had an unfavorable acute effect on endothelial function compared to decaffeinated coffee [40]. Impaired endothelial function is involved in the pathogenesis of atherosclerosis and cardiovascular disease [40].

The detrimental effect of espresso coffee suggested by our study findings could, at least in part, be due to its caffeine content and rapid consumption, which results in a high peak plasma concentration of caffeine. By contrast, with filtered coffee, the caffeine dose is similar but it is diluted in 140–200 ml of liquid and the beverage is drunk over several minutes. The detrimental effect of espresso coffee is also likely to vary with CYP1A2 genotype.

Although smoking is an established risk factor for cardiovascular disease, the association of coffee with CHD remained significant when adjusted for smoking status and intensity. We analyzed the non-smoker sub-cohort (46.5%) and found that coffee consumption was significantly associated with CHD: HRs: 1, 1.21 (95% CI 0.83,1.75); 1.47 (95% CI 1.04,2.09); and 1.70 (95% CI 1.15,2.51); P trend = 0.002 (data not shown). These findings indicate that the coffee-CHD association cannot be explained by smoking.

### Limitations

A limitation of the study is that coffee consumption and CHD risk factors were only assessed at baseline. We could not analyze the effect of decaffeinated coffee because this information was not available for the Ragusa and Napoli centers, and decaffeinated consumption was very low in the other centers. Furthermore we have no information about other major sources of caffeine, family history of CHD and hypertension, or sleep patterns or other psychological factors, so we cannot exclude some residual confounding by these variables not included in the multivariate model. Study subjects were volunteers recruited mainly among blood donors, women attending screening programs and their spouses, therefore they may not be fully representative of general population. Moreover, plasma lipid profile was available only for a random subsample which may not be representative of the cohort as a whole. Strengths of our study are its prospective design, and the fact that Italian coffee drinkers almost always drink coffee prepared by the espresso or mocha methods, thereby reducing confounding due to variation in preparation method.

## Conclusions

To conclude, we have found that consumption of more than 2 cups/day of Italian-style coffee is associated with significantly increased risk of CHD, but found no evidence that Italian-style coffee had any effect on plasma cholesterol or triglycerides. Further studies are required to determine whether drinking Italian-style coffee is causally associated with increased risk of CHD (and the association does arise, for example, because coffee consumption is marker of a lifestyle that increases that increases CHD risk); and also whether the risk is influenced by CYP1A2 genotype.
